# Elevated Circulating HMGB1 Levels as a Potential Biomarker for the Diagnosis and Therapy of Heart Failure: A Cross-Sectional Study

**DOI:** 10.31083/RCM49717

**Published:** 2026-06-17

**Authors:** Xiaoting Jiang, Xia Feng, Wen Liu, Shaolin Gong, Xiaoping Peng, Xiang Wang

**Affiliations:** ^1^Department of Cardiology, The First Affiliated Hospital, Jiangxi Medical College, Nanchang University, 330006 Nanchang, Jiangxi, China; ^2^Academician Workstation of Cardiovascular Innovative Materials, 330006 Nanchang, Jiangxi, China; ^3^Jiangxi Hypertension Research Institute, 330006 Nanchang, Jiangxi, China

**Keywords:** heart failure, high-mobility group box 1, biomarker, damage-associated molecular patterns, inflammation

## Abstract

**Background:**

High-mobility group box 1 (HMGB1), a damage-associated molecular pattern (DAMP), has increasingly been implicated in the pathogenesis of various cardiovascular diseases. This study aimed to elucidate the relationship between circulating HMGB1 levels and the development of heart failure (HF).

**Methods:**

This single-center cross-sectional study enrolled 412 patients presenting with chest tightness or pain at the Department of Cardiovascular Medicine, First Affiliated Hospital of Nanchang University, between January and July 2025. The relationship between HMGB1 and HF occurrence was evaluated using Spearman’s correlation analysis, logistic regression models, restricted cubic spline (RCS) plots, and receiver operating characteristic (ROC) curves. Subgroup analyses were also performed to assess the robustness of the findings.

**Results:**

Of the 412 enrolled patients, 343 were included in the final analysis after application of the exclusion criteria; 151 were diagnosed with HF, and 192 served as controls. Serum HMGB1 levels were significantly higher in the HF group than in the control group (1.53 ng/mL vs 0.93 ng/mL; *p* < 0.001). Spearman's correlation analysis revealed significant positive correlations between HMGB1 levels and left ventricular end-diastolic diameter (LVEDD), left atrial diameter (LAD), and the systemic inflammatory response index (SIRI), and a significant negative correlation with left ventricular ejection fraction (LVEF) (all *p* < 0.05). In the univariate logistic regression analysis, elevated HMGB1 levels were strongly associated with increased HF risk (odds ratio [OR] = 2.942, 95% confidence interval [CI]: 2.113–4.095; *p* < 0.001). This association remained significant in multivariable models after sequential adjustment for demographic, clinical, and laboratory covariates, with a fully adjusted OR of 2.273 (95% CI: 1.410–3.663; *p* < 0.001). Restricted cubic spline (RCS) analysis confirmed a linear dose–response relationship between HMGB1 levels and HF risk (*p* for nonlinearity = 0.174). ROC analysis showed that HMGB1 alone had good predictive value for HF (area under the curve [AUC] = 0.736), outperforming individual traditional markers. Furthermore, a combined model incorporating HMGB1, LVEF, LVEDD, LAD, and SIRI achieved superior predictive accuracy (AUC = 0.807). Finally, subgroup analyses showed that only sex exhibited a significant interaction, while interactions for all other variables were non significant interaction (* p* for interaction > 0.05), indicating that this relationship was robust and independent of major clinical variables.

**Conclusions:**

The levels of HMGB1 elevation were significantly related to the occurrence of HF, which may serve as a potential biomarker for the development of innovative therapeutic strategies in patients with heart failure.

## 1. Introduction

Heart failure (HF) is a multifaceted clinical syndrome defined by structural or functional cardiac dysfunction stemming from diverse etiologies, a condition that may progress to serious adverse cardiovascular events [1]. Despite a stabilization in incidence and mortality rates in developed nations, heart failure persists as a major global public health challenge with a high overall prevalence [2,3]. The results of the latest epidemiological survey in China indicate that the total number of prevalent cases of heart failure increased from 7.03 million in 1990 to 18.51 million in 2019, showing an increasing trend year by year, with a prevalence rate of 1.3% among residents aged 35 and older [4,5]. Characterized by high prevalence, mortality, and readmission rates, heart failure imposes a significant burden on public health, and the early detection and timely intervention of heart failure are crucial for improving patient outcomes and altering the disease trajectory [6].

The pathogenesis of heart failure is characterized by intricate crosstalk among multiple signaling pathways. Cardiac fibrosis, a key pathological feature of heart failure resulting from excessive collagen deposition, plays a crucial role in disease progression [7]. Accumulating evidence has further underscored that a spectrum of acute and chronic inflammatory responses serves as a central mediator in the pathogenesis of cardiac fibrosis [8]. A range of inflammatory mediators, including C-reactive protein and interleukins, have been consistently identified to exhibit a marked upregulation in patients with HF, and these factors are closely correlated with the long-term prognostic outcomes of HF patients [9,10].

HMGB1 was originally characterized as a highly conserved nuclear protein that is abundantly localized within the nucleosomes of eukaryotic organisms. Functionally, high mobility group box 1 (HMGB1) exerts multiple nuclear roles: it contributes to the stabilization of nucleosome assembly, facilitates the initiation and progression of gene transcription, and modulates the transcriptional activity of steroid hormone receptors [11]. Emerging evidence has demonstrated that HMGB1 also functions as a prototypical damage-associated molecular pattern (DAMP) molecule: upon cellular exposure to exogenous stress stimuli (e.g., oxidative stress, ischemia, or mechanical injury), it is translocated from the nucleus to cytoplasm, and subsequently secreted into the extracellular microenvironment [12]. Once extracellular, HMGB1 exerts its biological effects by engaging with pattern recognition receptors such as Toll-like receptors and the receptor for advanced glycation end products, and this signaling axis has been closely linked to the pathogenesis of a broad spectrum of inflammatory and metabolic disorders [13].

Although elevated HMGB1 levels have been documented in both human heart failure patients and murine models [14,15], comprehensive cross-sectional data detailing its levels and dynamic changes in the patient population remain scarce. This study aims to quantify serum HMGB1 levels in a well-characterized heart failure cohort to evaluate its potential as a clinically relevant diagnostic and therapeutic novel biomarker.

## 2. Materials and Methods

### 2.1 Study Population

This was a single-center cross-sectional study. A total of 412 patients with symptoms of chest tightness or chest pain in the Department of Cardiovascular Medicine at the First Affiliated Hospital of Nanchang University were selected as research subjects, and the study period was from January 2025 to July 2025. HF was diagnosed according to the 2021 European Society of Cardiology (ESC) guidelines. The diagnosis required the following criteria: manifested by typical clinical symptoms (e.g., dyspnea, fatigue) and/or signs (e.g., jugular venous distension, edema), supported by an N-terminal pro-B-type natriuretic peptide (NT-proBNP) level of ≥125 pg/mL, and confirmed by echocardiographic evidence of cardiac structural or functional abnormalities [16]; Exclusion criteria comprised: (1) Patients diagnosed with malignant neoplasms; (2) Patients with immune system disorders; (3) Patients with severe hepatorenal dysfunction; (4) Individuals with unavailable critical clinical data. This study was approved by the Ethics Committee of the First Affiliated Hospital of Nanchang University. Informed written consent was obtained from each participant prior to study enrollment. Applying these criteria, we enrolled 343 participants for subsequent analysis, yielding a final cohort of 151 patients in the heart failure group and 192 in the control group.

### 2.2 Clinical Data Collection

We prospectively collected comprehensive clinical data, including: (1) basic demographics: sex, age, body mass index, systolic and diastolic blood pressure; (2) laboratory investigations: triglycerides, total cholesterol (TC), low- and high-density lipoprotein cholesterol (LDL-C, HDL-C), white blood cell count (WBC), platelet count, neutrophil percentage, lymphocyte count, monocyte count, creatinine, uric acid, creatine kinase (CK), creatine kinase-myocardial band (CK-MB), fasting plasma glucose, alanine aminotransferase, aspartate aminotransferase, and N-terminal pro-B-type natriuretic peptide (NT-proBNP); (3) relevant medical history: hypertension, diabetes, smoking, and alcohol consumption; (4) echocardiographic parameters: left ventricular ejection fraction (LVEF), left atrial diameter (LAD), and left ventricular end-diastolic diameter (LVEDD), left ventricular diastolic function was evaluated using the mitral inflow E/A ratio and E/e′ ratio (E peak divided by the mean e′ peak of the interventricular septum and lateral mitral annulus); (5) medication use including angiotensin converting enzyme inhibitors (ACEIs)/angiotensin receptor blockers (ARBs), beta-blockers, calcium channel blockers, and diuretics.

### 2.3 Enzyme-Linked Immunosorbent Assay (ELISA)

Approximately 4 mL of blood was collected within 24 h of each patient’s admission by an experienced nurse after obtaining informed consent. Blood samples were allowed to clot and then centrifuged at 3500 ×g for 10 minutes to obtain serum. The serum supernatant was aliquoted and stored at –80 °C until further analysis. We measured serum HMGB1 levels using a commercial ELISA kit (Baipeng Biotechnology Co., Ltd., China, Shanghai, Catalog No. BP01600). According to the manufacturer, the assay has a detection range of 78–5000 pg/mL and a sensitivity of 40.7 pg/mL. A standard curve was generated using serial dilutions. All standards and samples were assayed in duplicate. Absorbance values were plotted against concentration and fitted using a four-parameter logistic (4PL) model, which consistently yielded a correlation coefficient (R^2^) >0.99. The intra-assay and inter-assay coefficients of variation are both <10%, ensuring good reproducibility of the measurements. The representative standard curves from five independent experiments were provided in the **Supplementary Materials** (**Supplementary Fig. 1**).

### 2.4 Statistical Analyses

All statistical analyses in the present study were performed with R software (version 4.2.3; R Foundation for Statistical Computing, Vienna, Austria). Continuous variables conforming to a normal distribution were presented as mean ± standard deviation and compared via independent-samples *t*-tests. Non-normally distributed continuous variables were summarized as median (interquartile range) and analyzed with Mann-Whitney U tests. Categorical variables were presented as frequencies (percentages) and assessed using chi-square tests.

Spearman’s rank correlation analysis was employed to evaluate associations between serum HMGB1 levels and LVEF, LVEDD, LAD, and systemic inflammatory response index (SIRI) in patients. Plotted receiver operating characteristic (ROC) curves to assess the diagnostic performance of HMGB1 for heart failure, with quantification of the AUC. To investigate the potential non-linear relationships between the serum HMGB1 levels and HF, restricted cubic spline (RCS) plots were utilized. Factors significant in univariate models were entered into a series of multivariate logistic regression models with sequential adjustment to determine those independently associated with heart failure. Finally, subgroup analyses were performed, subgrouped by sex, age, smoking, drinking, hypertension, and diabetes, to explore other potential confounding factors affecting the relationship between HMGB1 and heart failure.

## 3. Results

### 3.1 Baseline Characteristics of the Study Population

**Fig. 1.**
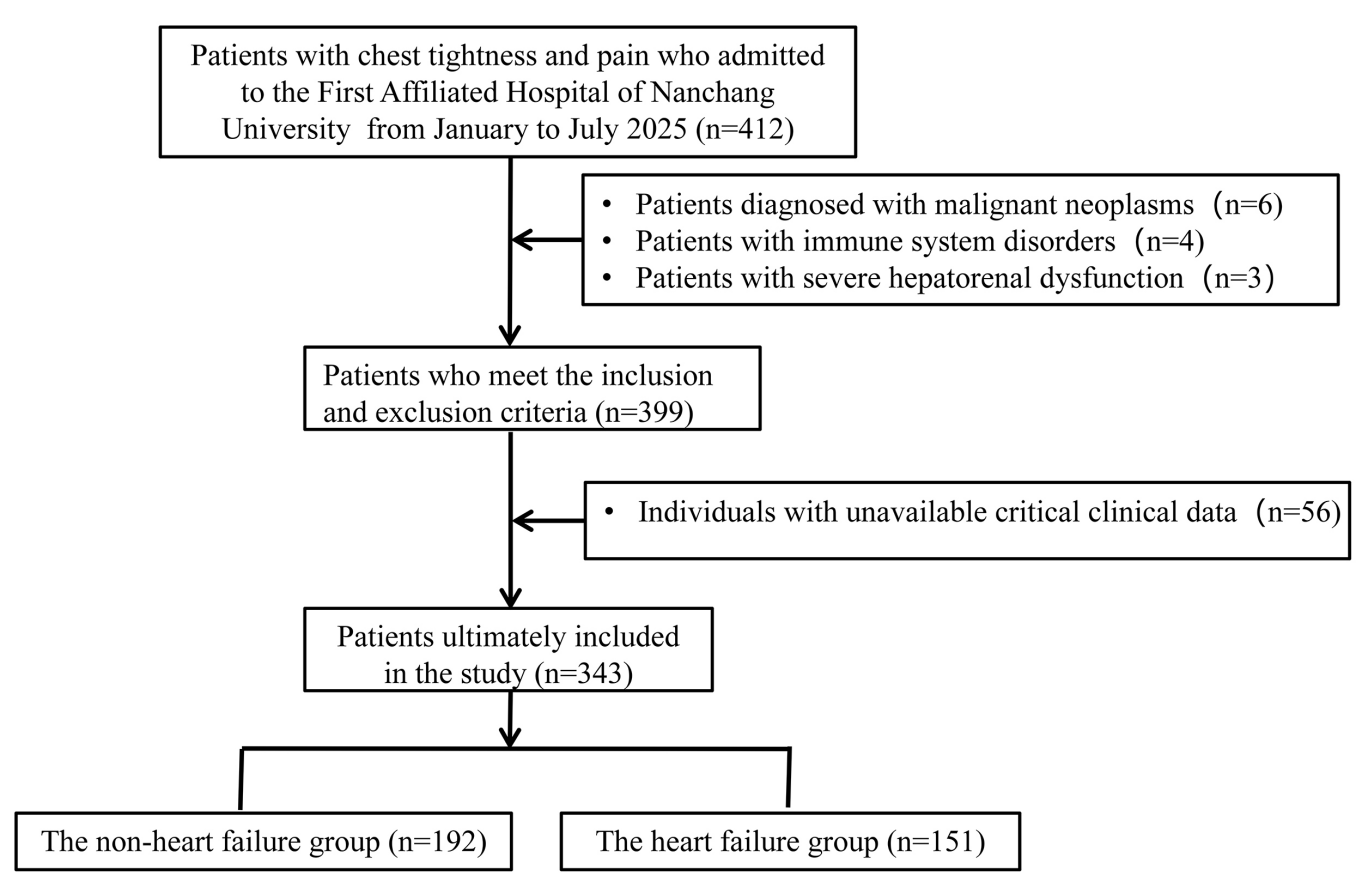
**Flow diagram illustrating the screening, enrollment, and exclusion processes of participants**.

A total of 412 patients were enrolled in the study. According to the inclusion and exclusion criteria, ultimately, we enrolled 343 participants for subsequent analysis, yielding a final cohort of 151 patients in the heart failure group and 192 in the control group (Fig. 1).

A total of 343 participants were recruited for the present analysis, among whom 219 (63.85%) were male, with a median age of 65 years. According to serum HMGB1 levels, the study population was stratified into three tertiles: Tertile 1: 0 < HMGB1 ≤ 0.896 ng/mL; Tertile 2: 0.896 < HMGB1 ≤ 1.472 ng/mL; and Tertile 3: HMGB1 >1.472 ng/mL. Baseline characteristics were compared across the three tertiles, and the results are presented in Table 1.

**Table 1.** **Baseline characteristics of study population by tertiles of the HMGB1**.

Variables		Total (n = 343)	Tertile 1 (n = 114)	Tertile 2 (n = 115)	Tertile 3 (n = 114)	*p*
Age, years		65.00 (57.00, 71.00)	58.00 (50.00, 67.00)	65.00 (59.50, 71.00)	69.5 (62.25, 75.00)	<0.001
Male, n (%)		219 (63.85)	67 (58.77)	74 (64.35)	78 (68.42)	0.314
BMI, kg/m^2^		24.26 (21.92, 26.42)	24.79 (22.50, 26.88)	24.00 (21.60, 26.08)	23.98 (21.11, 26.64)	0.146
Smoking, n (%)		142 (41.40)	42 (36.84)	45 (39.13)	55 (48.25)	0.181
Drinking, n (%)		80 (23.32)	31 (27.19)	23 (20.00)	26 (22.81)	0.431
Hypertension, n (%)		197 (57.43)	53 (46.49)	63 (54.78)	81 (71.05)	<0.001
Diabetes, n (%)		80 (23.32)	16 (14.04)	23 (20.00)	41 (35.96)	<0.001
Heart Failure, n (%)		151 (44.02)	24 (21.05)	49 (42.61)	78 (68.42)	<0.001
CAD, n (%)		228 (66.47)	73 (64.04)	74 (64.35)	81 (71.05)	0.447
AF, n (%)		7 (2.04)	1 (0.88)	3 (2.61)	3 (2.63)	0.707
SBP, mmHg		129.00 (115.00, 144.00)	126.00 (114.00, 137.75)	131.00 (115.00, 144.50)	132.00 (117.25, 145.00)	0.054
DBP, mmHg		72.00 (63.00, 82.00)	73.00 (65.00, 81.75)	74.00 (65.00, 82.00)	67.50 (63.00, 80.75)	0.178
HR, bpm		81.00 (72.00, 89.00)	81.00 (72.00, 88.00)	78.00 (72.00, 89.00)	81.50 (71.25, 89.00)	0.814
Drug utilization						
	ACEI/ARB, n (%)	88 (25.66)	12 (10.53)	33 (28.70)	43 (37.72)	<0.001
	Beta-blockers, n (%)	93 (27.11)	17 (14.91)	32 (27.83)	44 (38.60)	<0.001
	CCB, n (%)	50 (14.58)	9 (7.89)	21 (18.26)	20 (17.54)	0.046
	Diuretics, n (%)	31 (9.04)	3 (2.63)	12 (10.43)	16 (14.04)	0.009
Laboratory indicators						
	White blood cells, 10^9^/L	6.25 (5.18, 7.49)	6.51 (5.19, 7.60)	5.89 (5.03, 7.04)	6.41 (5.42, 7.99)	0.027
	ALT, U/L	20.30 (15.00, 28.70)	20.65 (15.83, 28.00)	20.15 (15.35, 30.17)	19.70 (13.90, 28.40)	0.401
	AST, U/L	21.80 (18.30, 27.80)	21.20 (18.40, 25.58)	21.90 (18.10, 27.00)	22.60 (17.80, 31.20)	0.515
	Creatinine, μmol/L	71.75 (60.80, 86.30)	65.55 (60.05, 75.30)	70.80 (57.55, 82.15)	85.80 (70.80, 109.20)	<0.001
	Uric acid, μmol/L	332.70 (267.88, 398.77)	301.65 (252.45, 365.52)	325.90 (258.70, 380.65)	365.90 (310.20, 473.80)	<0.001
	CK, U/L	99.95 (72.27, 135.43)	101.50 (72.40, 130.30)	102.05 (79.80, 135.40)	96.80 (70.80, 139.00)	0.683
	CK-MB, U/L	17.10 (14.30, 22.10)	17.10 (14.70, 21.90)	16.40 (14.30, 19.50)	18.40 (14.10, 24.40)	0.183
	LDH, U/L	221.50 (191.62, 262.55)	219.6 (189.15, 261.02)	213.30 (189.75, 242.75)	231.60 (202.40, 287.30)	0.022
	SII	570.18 (392.56, 856.19)	614.57 (419.09, 837.92)	510.89 (365.66, 805.95)	598.05 (418.88, 1004.86)	0.067
	SIRI	1.07 (0.72, 1.53)	1.00 (0.70, 1.42)	0.97 (0.60, 1.38)	1.30 (0.96, 1.90)	<0.001
	FPG, mmol/L	6.04 (5.02, 8.11)	5.84 (4.94, 7.15)	6.05 (5.21, 7.09)	6.46 (5.07, 9.70)	0.046
	Total cholesterol, mmol/L	4.23 (3.47, 5.12)	4.55 (3.60, 5.27)	4.28 (3.67, 5.14)	3.93 (3.21, 4.80)	0.004
	Triglycerides, mmol/L	1.51 (1.13, 2.27)	1.54 (1.15, 2.35)	1.46 (1.08, 2.46)	1.46 (1.17, 2.10)	0.805
	High-density lipoprotein, mmol/L	1.02 (0.88, 1.22)	1.05 (0.92, 1.21)	1.03 (0.90, 1.25)	0.98 (0.85, 1.16)	0.021
	Low-density lipoprotein, mmol/L	2.35 (1.73, 2.99)	2.55 (1.82, 3.13)	2.36 (1.85, 2.98)	2.15 (1.59, 2.77)	0.015
	NT-pro BNP, ng/L	131.10 (106.50, 658.00)	108.70 (74.50, 130.00)	130.90 (112.50, 521.00)	532.00 (130.25, 1343.10)	<0.001
Echocardiography information						
	E/A	0.75 (0.65, 0.91)	0.76 (0.67, 1.11)	0.75 (0.66, 0.94)	0.72 (0.62, 0.86)	0.227
	E/e′	9.30 (7.73, 11.45)	8.80 (7.43, 10.89)	9.33 (8.06, 11.29)	10.00 (8.00, 12.67)	0.009
	LVEF, %	60.00 (59.00, 63.00)	61.00 (60.00, 63.00)	60.00 (59.50, 63.50)	60.00 (55.00, 63.00)	0.002
	LAD, mm	33.00 (30.00, 37.00)	32.00 (29.00, 35.00)	34.00 (31.00, 37.00)	35.00 (30.25, 39.00)	<0.001
	LVEDD, mm	46.00 (43.00, 49.00)	45.00 (43.00, 48.00)	47.00 (43.00, 50.00)	46.00 (44.00, 51.75)	0.024

Tertile 1: 0 < HMGB1 ≤ 0.896 ng/mL; Tertile2: 0.896 ng/mL < HMGB1 ≤ 1.472 ng/mL; Tertile3: HMGB1 >1.472 ng/mL. BMI, body mass index; CAD, coronary artery disease; AF, atrial fibrillation; HR, heart rate; SBP, systolic blood pressure; DBP, diastolic blood pressure; ACEI, angiotensin converting enzyme inhibitor; ARB, angiotensin receptor blocker; CCB, Calcium channel blockers; FPG, fasting plasma glucose; ALT, alanine amino transferase; AST, aspartate amino transferase; CK, creatine kinase; CK-MB, creatine kinase-myocardial Band; LDH, lactate dehydrogenase; SII, Systemic immune-inflammation index; SIRI, systemic inflammatory response index; LVEF, left ventricular ejection fraction; LAD, left atrial diameter; LVEDD, left ventricular end-diastolic diameter; HMGB1, high mobility group box 1.

**Fig. 2.**
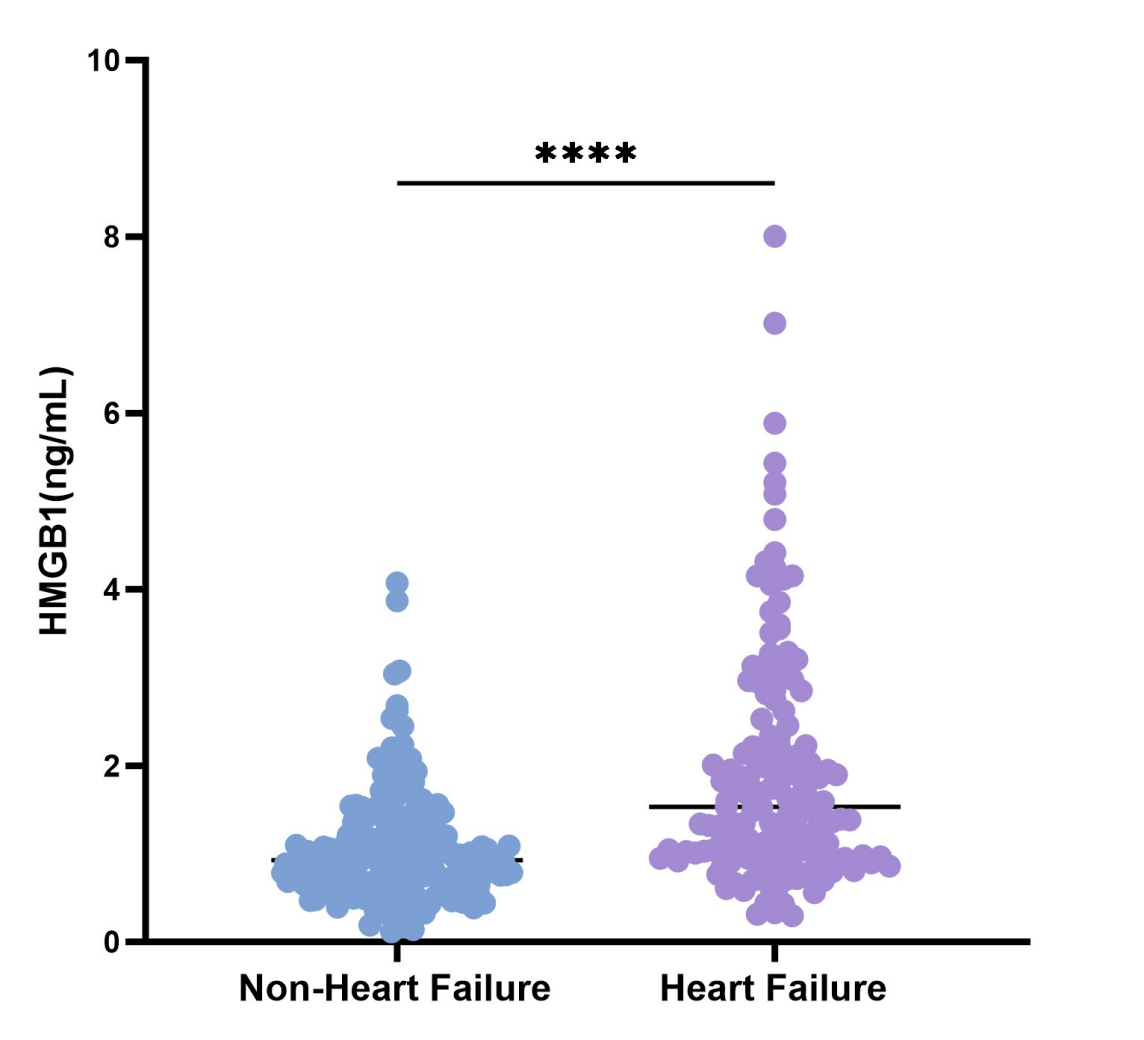
**HMGB1 levels in non-heart failure and heart failure groups**. ****p < 0.0001.

As detailed in Table 1, hypertension and diabetes mellitus increased with elevated serum HMGB1 levels, accompanied by a higher proportion of patients receiving angiotensin-converting enzyme inhibitors/angiotensin receptor blockers (ACEIs/ARBs), beta-blockers, calcium channel blockers, and diuretics. Patients in Tertile 3 had a median age of 70 years, which was significantly higher than the overall median age of the study population. With increasing HMGB1 levels, subjects exhibited higher levels of WBC, SIRI, lactate dehydrogenase (LDH), Creatinine, Uric acid, and NT-proBNP, as well as lower levels of HDL-C, LDL-C and TC. Regarding echocardiographic parameters, Tertile 3 was also associated with larger LAD, and E/e′ compared with the other two tertiles. Among the 151 patients with heart failure, 24 (15.89%), 49 (32.45%), and 78 (51.66%) were distributed in Tertile 1, Tertile 2, and Tertile 3 of serum HMGB1 levels, respectively. Additionally, this pattern was robustly confirmed by direct comparison, which showed that median HMGB1 levels were significantly higher in the heart failure group than in controls (1.53 [1.01, 2.34] ng/mL vs. 0.93 [0.65, 1.35] ng/mL; *p* < 0.0001) (Fig. 2).

### 3.2 Correlation Analysis Between serum HMGB1 Levels and LVEF, LVEDD, LAD, and SIRI

**Fig. 3.**
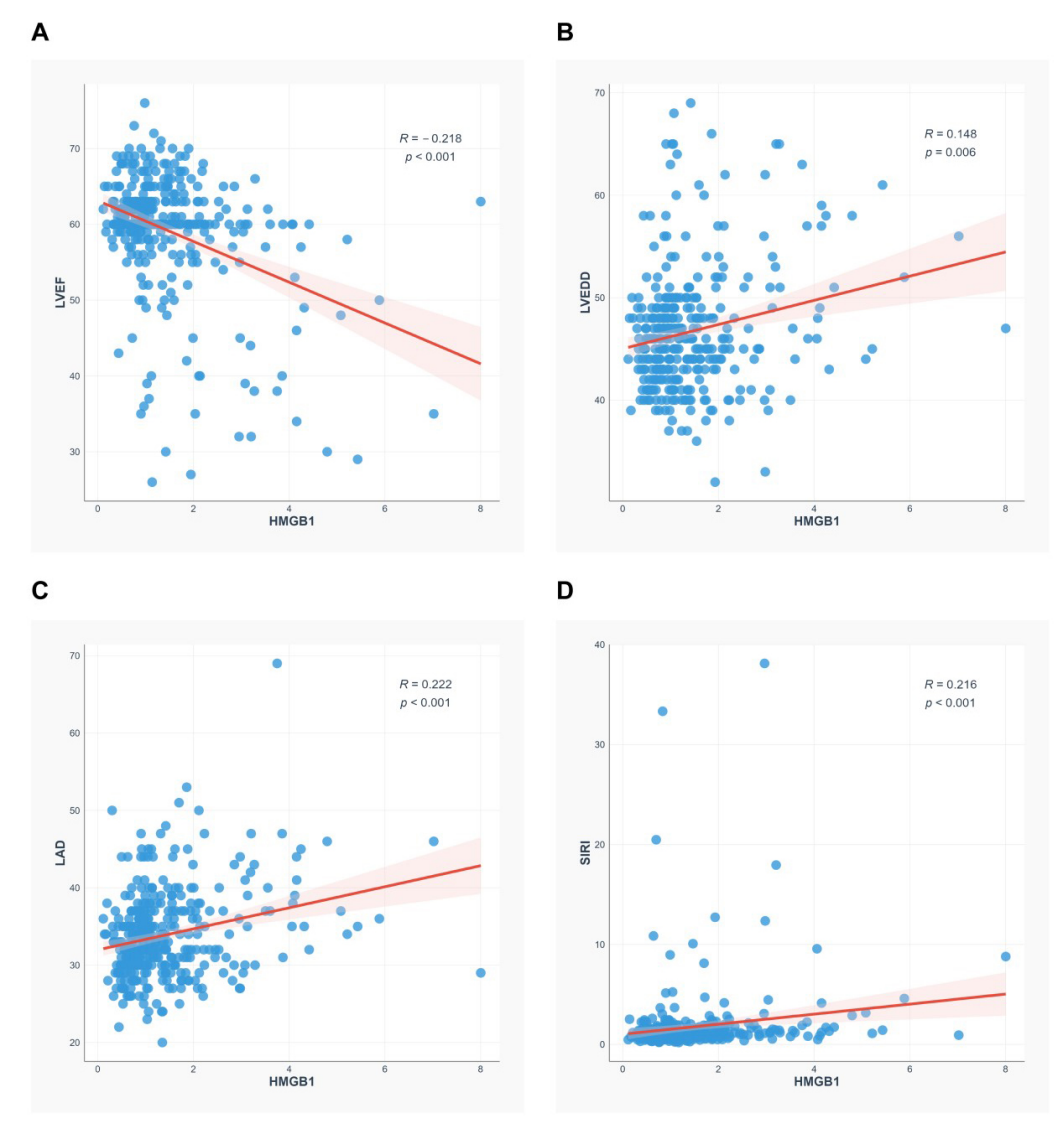
**Scatter plot of correlation between HMGB1 levels and LVEF, LVEDD, LAD, SIRI**. (A–D) Correlations between HMGB1 levels and LVEF (A), LVEDD (B), LAD (C), and SIRI (D).

Spearman correlation analysis was performed to assess the relationships between serum HMGB1 levels and cardiac function parameters (LVEF, LVEDD, LAD) as well as SIRI. HMGB1 levels were significantly positively correlated with LVEDD, LAD, and SIRI (*p* < 0.05), such that these indices increased progressively with the elevation of HMGB1 levels. Conversely, LVEF, a core indicator reflecting cardiac systolic function, was significantly negatively correlated with HMGB1 levels (*p *< 0.001), displaying a gradual decreasing trend as HMGB1 levels rose (Fig. 3).

### 3.3 Association Between Serum HMGB1 Levels and Occurrence of Heart Failure

**Fig. 4.**
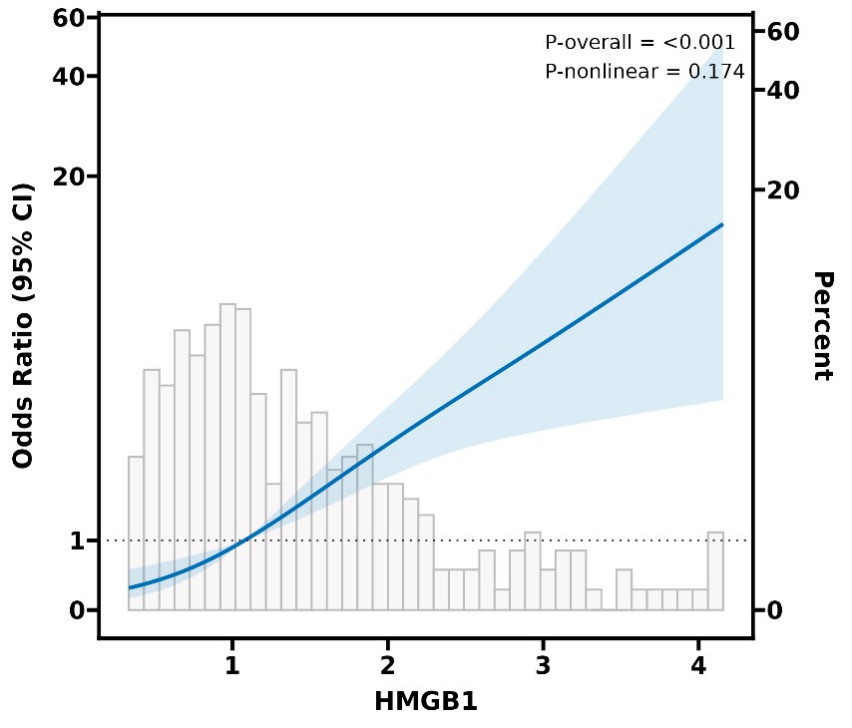
**RCS plot of HMGB1 levels and the occurrence of heart failure**. RCS, restricted cubic spline.

Elevated serum HMGB1 was found to have a significant positive association with HF risk via univariate logistic regression analysis (OR = 2.942, 95% CI: 2.113–4.095, *p* < 0.001; **Supplementary Table 1**). This association remained statistically significant across sequential multivariate logistic regression models (Table 2). Model 2, adjusted for demographic covariates including age, gender, and BMI, yielded an adjusted OR of 2.685 (95% CI: 1.914–3.766, *p* < 0.001). Model 3, which further incorporated lifestyle and comorbidity factors: smoking status, alcohol consumption, hypertension, and diabetes based on Model 2 adjustments, confirmed that serum HMGB1 remained an independent predictor of HF, with an OR of 2.715 (95% CI: 1.905–3.869, *p* < 0.001). In the fully adjusted Model 4, incorporating comprehensive laboratory parameters, coronary artery disease (CAD) and atrial fibrillation (AF) in addition to the aforementioned covariates, serum HMGB1 remained significantly associated with an increased risk of HF onset (OR = 2.273, 95% CI: 1.410–3.663, *p* < 0.001), further supporting its role as an independent risk factor. Furthermore, a significant dose-response relationship was observed: compared with the lowest tertile of HMGB1, the highest tertile conferred a 5.904-fold elevated risk (95% CI: 2.416–14.429, *p* < 0.001). Collectively, these findings demonstrate that serum HMGB1 is a robust independent risk factor for HF. We employed RCS to explore the potential nonlinear relationship between serum HMGB1 levels and heart failure incidence. After appropriate data trimming to exclude outliers, the analysis revealed a significant linear association between HMGB1 and heart failure risk (*p* for nonlinearity = 0.174). The dose-response relationship demonstrated a monotonically increasing trend in heart failure incidence with rising HMGB1 levels (Fig. 4).

**Table 2.** **Effect of HMGB1 level on heart failure in different models**.

Variables	Model 1		Model 2		Model 3		Model 4	
	OR (95% CI)	*p*	OR (95% CI)	*p*	OR (95% CI)	*p*	OR (95% CI)	*p*
HMGB1	2.942 (2.113~4.095)	<0.001	2.685 (1.914~3.766)	<0.001	2.715 (1.905~3.869)	<0.001	2.273 (1.410~3.663)	<0.001
HMGB1 Tertile								
1	1.000 (Reference)		1.000 (Reference)		1.000 (Reference)		1.000 (Reference)	
2	2.784 (1.555~4.985)	<0.001	2.379 (1.291~4.385)	0.005	2.377 (1.280~4.417)	0.006	1.891 (0.864~4.136)	0.111
3	8.125 (4.464~14.789)	<0.001	6.344 (3.289~12.236)	<0.001	6.252 (3.155~12.391)	<0.001	5.904 (2.416~14.429)	<0.001

Model 1: Unadjusted; Model 2: Adjusted for gender, age, and BMI; Model 3: Adjusted for gender, age, BMI, smoking, drinking, hypertension and diabetes; Model 4: Adjusted for gender, age, BMI, smoking, drinking, hypertension, diabetes, CAD, AF and laboratory indicators (neutrophil percentage, LDH, total cholesterol, SIRI, triglycerides, high-density lipoprotein cholesterol, CK-MB, low-density lipoprotein) and echocardiography information (LVEF, LVEDD, LAD, E/A and E/e′). OR, odds ratio; CI, confidence interval.

### 3.4 Predictive Performance of HMGB1 and Other Biomarkers for Heart Failure

The predictive performance of serum HMGB1 and other clinical parameters for heart failure was evaluated using ROC analysis. As summarized in Table 3, the area under the curve (AUC) for HMGB1 alone was 0.736 (95% CI: 0.684–0.790). This was comparable to the AUC of LVEF, which was 0.714 (95% CI: 0.657–0.771). In contrast, the predictive values of LVEDD, LAD, and SIRI were lower, with AUC of 0.650 (95% CI: 0.591–0.711), 0.680 (95% CI: 0.622–0.738), and 0.615 (95% CI: 0.555–0.675).

**Table 3.** **ROC analysis of individual and combined indicators to predict the occurrence of heart failure**.

Characteristic		AUC	95% CI	Specificity	Sensitivity	Cut off
**Individual indicators**						
	HMGB1	0.736	(0.684, 0.790)	0.609	0.741	1.311
	LVEF	0.714	(0.657, 0.771)	0.490	0.865	58.500
	LVEDD	0.650	(0.591, 0.711)	0.510	0.757	47.500
	LAD	0.680	(0.622, 0.738)	0.490	0.812	35.500
	SIRI	0.615	(0.555, 0.675)	0.616	0.598	
**Combined indicators**						
	HMGB1+LVEF	0.781	(0.731, 0.831)	0.563	0.899	-
	HMGB1+LVEDD	0.768	(0.718, 0.818)	0.570	0.847	-
	HMGB1+LAD	0.771	(0.721, 0.822)	0.616	0.825	-
	HMGB1+SIRI	0.747	(0.694, 0.799)	0.768	0.598	-
	All	0.807	(0.760, 0.853)	0.629	0.852	-

ROC, receiver operating characteristic.

**Fig. 5.**
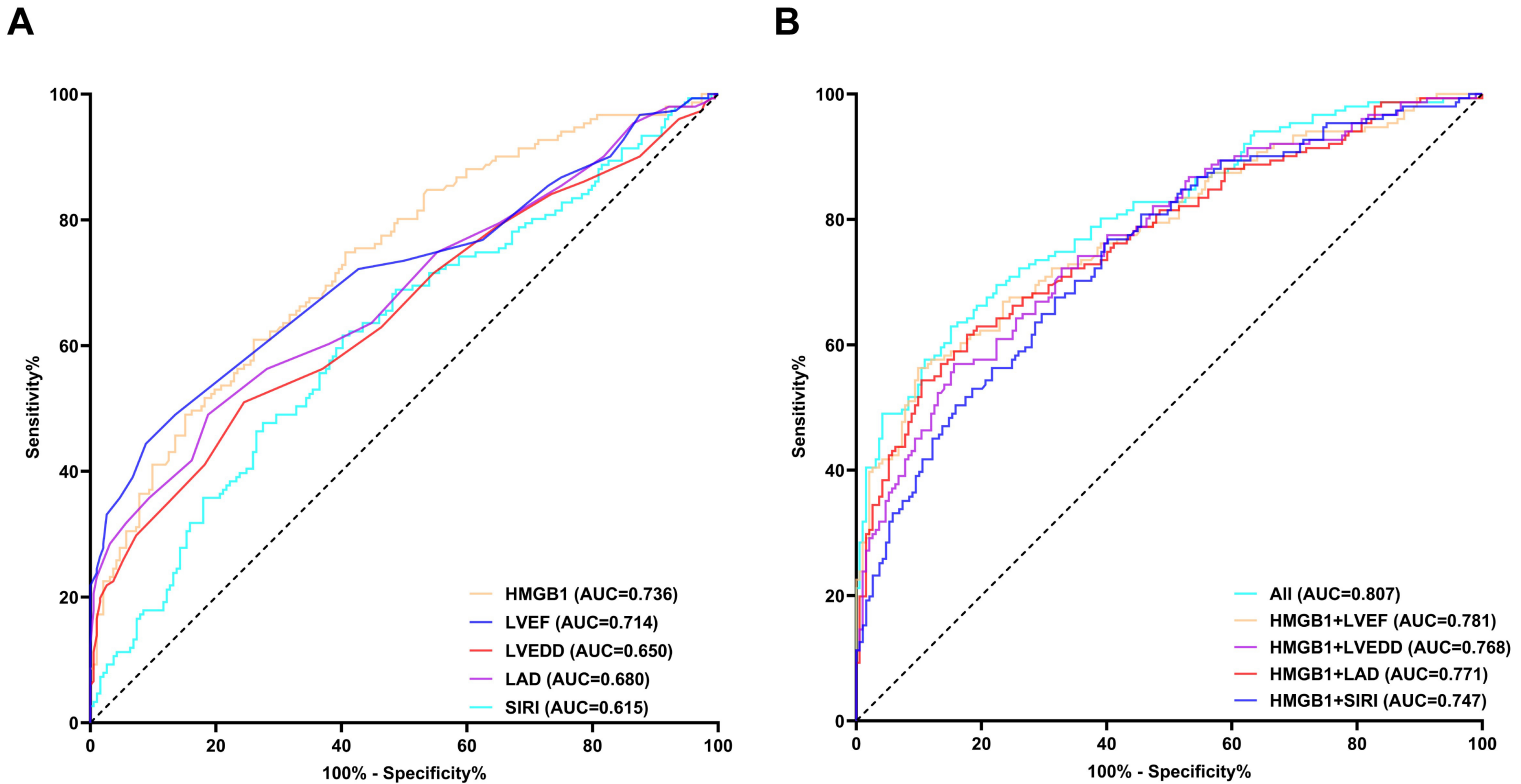
**ROC curve analyses of HMGB1 and the combined indices in predicting heart failure**. (A) shows the ROC curve of the single indicator, (B) presents the ROC curve for HMGB1 combined with other indicators.

We further assessed the performance of combined biomarker panels. The integration of HMGB1 with the other parameters significantly enhanced the predictive accuracy. All combination models achieved AUCs greater than 0.700. Specifically, the combination of HMGB1 with LVEF, LVEDD, LAD, and SIRI yielded the highest predictive power, with an AUC of 0.807 (95% CI: 0.760–0.853) (Fig. 5).

### 3.5 Subgroup Analyses and Interaction Effects of HMGB1 on Heart Failure Risk

**Fig. 6.**
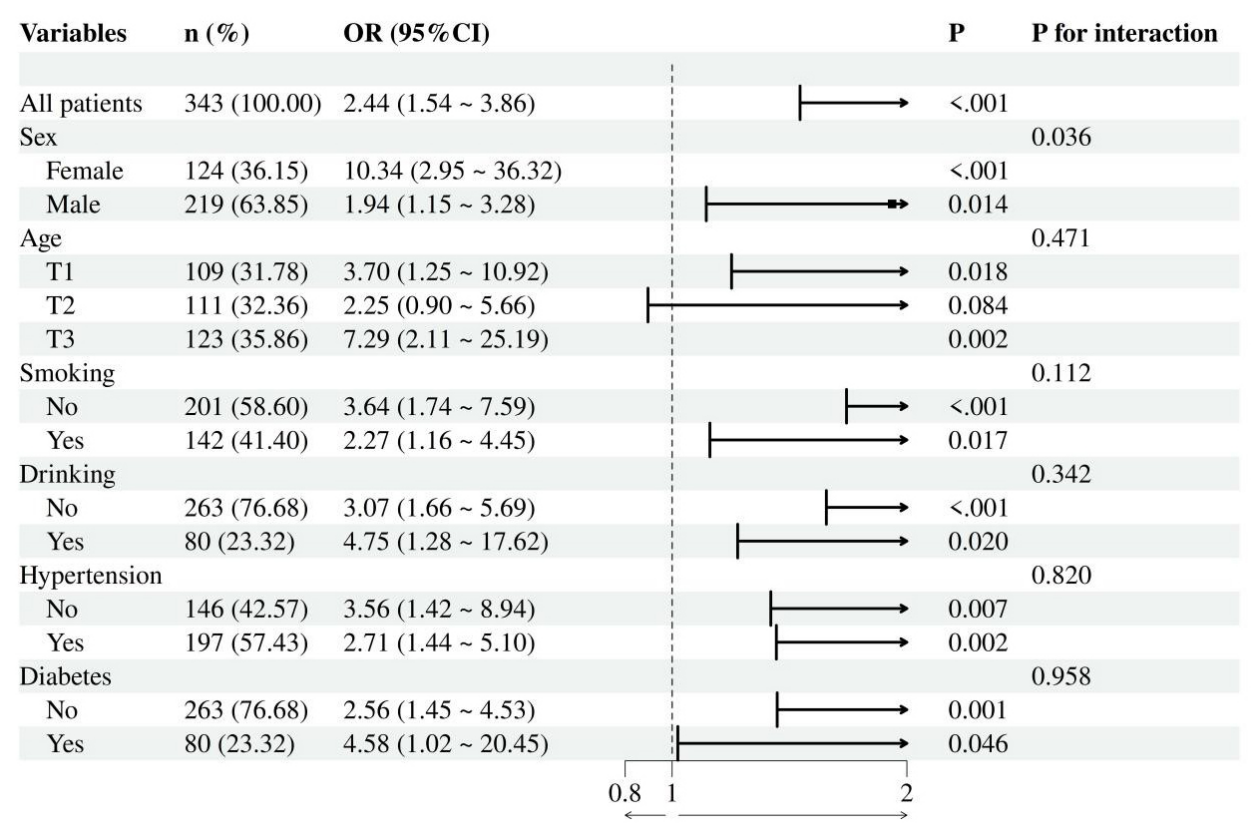
**Forest plot of the effect of HMGB1 levels on heart failure in different subgroup analyses**. Age was stratified by tertile groups; T1: Age ≤60; T2: 60 < Age ≤ 69; T3: Age >69. Adjusted for gender, age, BMI, smoking, drinking, hypertension and diabetes and laboratory indicators (neutrophil percentage, SIRI, CK-MB, LDH, total cholesterol, triglycerides, high-density lipoprotein cholesterol, low-density lipoprotein) and echocardiography information (LVEF, LVEDD and LAD).

To assess the consistency of the association between HMGB1 and heart failure, we conducted subgroup analyses stratified by gender, age, hypertension, diabetes, smoking, and alcohol consumption. Interaction effects were evaluated using likelihood ratio tests. Odds ratios (OR) with 95% confidence intervals (CIs) for the HMGB1-HF association were calculated for each subgroup (Fig. 6), and the association remained statistically significant with confounder adjustment, indicating the consistent independent association of HMGB1 with HF risk across subgroups and confirming the robustness of this relationship.

## 4. Discussion

Our study aimed to investigate the association between serum HMGB1 and HF. Our key findings revealed that serum HMGB1 levels were markedly elevated in patients with HF compared to the control group (1.53 ng/mL vs. 0.93 ng/mL, *p* < 0.001), and exhibited a marked dose-dependent correlation with HF risk. Additionally, HMGB1 demonstrated notable predictive value for HF, with an AUC of 0.736, and multivariable logistic regression analyses further confirmed HMGB1 as a novel biomarker for HF. The 30% missing rate of high-sensitivity C-reactive protein (hs-CRP) data in actual collection precluded valid statistical analysis, so we used SIRI as an alternative inflammatory biomarker. Notably, our findings confirmed a positive HMGB1 and SIRI correlation, and their combination improved diagnostic performance. This is consistent with prior studies establishing the prognostic value of composite inflammatory markers in patients with heart failure with reduced ejection fraction, particularly those undergoing device therapy [17], thereby adding to the growing body of evidence for the inflammatory pathogenesis underlying heart failure.

Heart failure, as the end stage of multiple cardiovascular diseases, is characterized by complex structural, functional, and molecular pathologies that collectively impair cardiac output [18]. Among the key drivers of HF progression, inflammation and myocardial fibrosis have been increasingly recognized as central pathological processes [19], which are mediated through intricate interactions between macrophages and fibroblasts via paracrine signaling [20]. In this context, high mobility group protein 1, a DAMP, has garnered increasing interest for its potential role in HF pathogenesis. We also acknowledge that a single inflammatory indicator cannot fully explain the complexity of HF. Currently, numerous studies integrate multiple indices to predict heart failure, thereby enhancing the persuasiveness of their findings. For instance, a previous study validated albumin’s prognostic role in HF patients treated with cardiac resynchronization therapy defibrillators [21], as albumin integrates nutritional and inflammatory status, it effectively improves risk stratification. Consequently, various composite indices based on albumin have also been shown to predict long-term mortality in HF patients [22,23]. Similarly, the combination of HMGB1 and SIRI in our study strengthens the inflammatory mediating role of HMGB1, further reinforcing the credibility of such multi-index integration strategies.

Multiple studies have shown that macrophages secrete substantial HMGB1 into the cytoplasm or extracellular space upon external stimulation, mediating various diseases via activation of downstream signaling pathways [24,25]. Notably, elevated HMGB1 levels significantly influence prognosis and long-term recovery in Intensive Care Unit (ICU) patients [26], highlighting the clinical value of early HMGB1 modulation. In cardiovascular diseases, HMGB1 has been increasingly implicated: a study of 258 patients found significantly elevated serum HMGB1 within 24 h of onset for unstable angina and non-ST-segment elevation, with follow-up levels independently predicting cardiovascular mortality [27]; another study identified HMGB1 as a biomarker for acute myocardial infarction regardless of HF status [28]; and a prospective study demonstrated elevated HMGB1 in HF patients, with levels independently predicting 12-month mortality [29]. While previous studies have identified HMGB1 as a serum biomarker, most were conducted in cohorts of patients with MI and featured relatively small sample sizes. The novelty of our study resides in its specific focus on patients presenting with chest pain: we systematically assessed the diagnostic value of HMGB1 for HF in this distinct population and established its optimal diagnostic cutoff value via RCS and ROC curve analyses. We found that after sequential adjustment for demographic, clinical, and laboratory covariates, HMGB1 remained independently associated with HF (fully adjusted OR = 2.273, 95% CI: 1.410–3.663, *p* < 0.001), confirming robustness independent of major confounders. Notably, unlike some traditional cardiovascular biomarkers such as NT-proBNP that are more closely linked to hemodynamic changes, HMGB1 reflects core inflammatory and fibrotic pathological processes of HF, complementing existing markers for more comprehensive risk stratification [30]. But the role of HMGB1 as an inflammatory DAMP in the pathogenesis of HF remains to be further explored: it is currently unclear whether HMGB1 is a causal factor, a secondary outcome of heart failure, or both [31]. This unresolved controversy leads to the inherent limitations of a single serum HMGB1 test, restricting the clinical application of our research findings. While our study’s sample size sufficiently validated this association, the study design limited our ability to establish temporal causality and long-term prognostic value; we plan to address this gap through prospective follow-up studies to further clarify HMGB1’s utility in HF management.

Our correlation analysis demonstrated that elevated HMGB1 levels were significantly associated with adverse cardiac remodeling, as reflected by increased LAD and LVEDD, coupled with a decrease in LVEF. These structural and functional impairments are well-established hallmarks of heart failure severity [32]. Concurrently, HMGB1 showed a positive correlation with the SIRI, reinforcing its pro-inflammatory role [33]. Although multiple multivariate scoring systems and biochemical biomarkers are currently available for predicting heart failure progression [34], our study highlights HMGB1’s unique advantage, achieving a predictive AUC of 0.736 for heart failure onset. Furthermore, a combined model integrating HMGB1 with LVEF, LVEDD, LAD, and SIRI demonstrated superior predictive accuracy (AUC = 0.807). In our study, patients at baseline exhibited mild left ventricular diastolic dysfunction, as shown by the mitral inflow E/A ratio and E/e′ ratio, which is a typical feature of heart failure. Notably, the E/e′ ratio rather than the E/A ratio showed significant differences between groups, highlighting the higher sensitivity of the former to diastolic load. After adjusting for these diastolic function indices, the association between HMGB1 and heart failure remained robust, indicating that its pathogenic effects are independent of the baseline diastolic dysfunction. However, we acknowledge the study limitation is the inability to directly compare HMGB1 with the gold-standard NT-proBNP in ROC and multivariate analyses [35], due to extensive missing NT-proBNP data within 24 hours of admission and exclusion of delayed data to avoid early treatment bias. The New York Heart Association classification, a key clinical parameter that categorizes heart failure severity based on symptoms and physical activity, was not available due to the retrospective, real-world design of this study. Given its critical role in heart failure assessment [36], future research will systematically incorporate these indicators.

A key finding of our study was the significant positive correlation between HMGB1 levels and advancing age. Patients in the highest HMGB1 tertile had a median age of 70 years, substantially older than the cohort average of 65 years. Current research indicates that one hallmark of aging is the gradual accumulation of chronic, low-level systemic inflammation [37]. This association is biologically plausible given that aging is characterized by chronic low-grade inflammation driven by senescent cells releasing pro-inflammatory mediators via the senescence-associated secretory phenotype (SASP) [37,38]. The age-related rise in HMGB1 suggests that aging may amplify its detrimental effects on cardiac function. Importantly, the consistent association between HMGB1 and HF across all age subgroups underscores its robust predictive value independent of aging-related inflammatory changes. Moreover, the influence of renal function on HMGB1 levels represents a significant consideration. Studies indicate that impaired kidney function can reduce the clearance capacity of HMGB1, leading to its accumulation in plasma [39]. Furthermore, in patients with chronic kidney disease, serum HMGB1 levels are markedly elevated and closely associated with disease progression and prognosis [24]. However, in the present study, the potential impact of this factor was mitigated by the exclusion of patients with severe kidney disease during participant enrollment.

As noted above, our study has several limitations that should be acknowledged, along with corresponding future improvements. First, despite the relatively large sample size supporting the assessment of serum HMGB1’s association with HF, the single-center design may limit the generalizability of the results. Second, the control group consisted of patients with chest symptoms but without concurrent HF. If these patients also had other cardiovascular diseases that could elevate HMGB1 levels, this might introduce bias; subsequent research should include age and sex matched healthy controls to minimize confounding. Third, HMGB1 levels were only measured within 24 hours of hospitalization, precluding the tracking of dynamic changes throughout disease progression. The longitudinal monitoring of HMGB1 during treatment and follow-up would help clarify its role in disease trajectories. Additionally, a potential limitation of this study is that cardiac function parameters such as NT-proBNP and LVEF may have been influenced by concomitant medications in our patient cohort, which could introduce bias into the results [40]. To address these questions, our team plans to initiate a prospective follow-up study. This study will involve the systematic collection of comprehensive clinical data, including detailed HF etiology, disease duration, and other potential confounders. With these data, we will perform more rigorous multivariable statistical adjustments. Furthermore, a study focusing on predicting myocardial injury in elderly patients demonstrated that machine learning could enhance prediction accuracy [41]. In future research, we may refer to the machine learning approach employed in this study to further optimize our model by capturing nonlinear interactions. By integrating the interactions between HMGB1 and other inflammatory indicators, we will further verify the association between HMGB1 and HF, explore its potential causal role, and analyze key clinical outcomes, thereby endowing this study with greater clinical practical value.

## 5. Conclusions

Our study investigated the significance of HMGB1 in heart failure, providing predictive value that complements current clinical parameters. As a readily measurable circulating biomarker linked to fundamental disease mechanisms, HMGB1 holds significant promise for improving HF risk assessment and may serve as a potential biomarker for the development of innovative therapeutic strategies.

## Data Availability

The datasets used and analyzed during the current study can be obtained from the corresponding author upon reasonable request.
