# Supplementary material for: Elevated Circulating HMGB1 Levels as a Potential Biomarker for the Diagnosis and Therapy of Heart Failure: A Cross-Sectional Study
Source: Rev Cardiovasc Med. 2026 Jun 17;27(6):49717. doi: 10.31083/RCM49717 (PMC13339188; doi:10.31083/RCM49717)
Supplement: Supplementary file 1 [file 2153-8174-27-6-49717-s1.zip › Supplementary Material.docx]

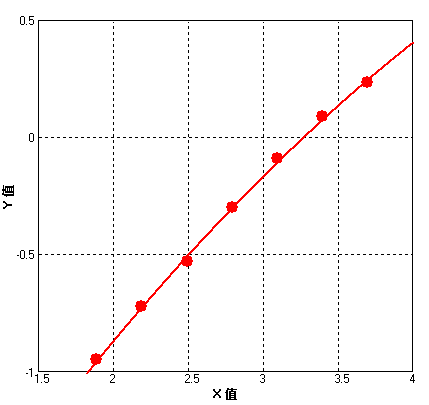

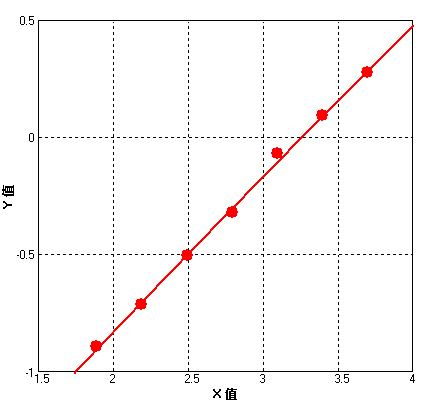

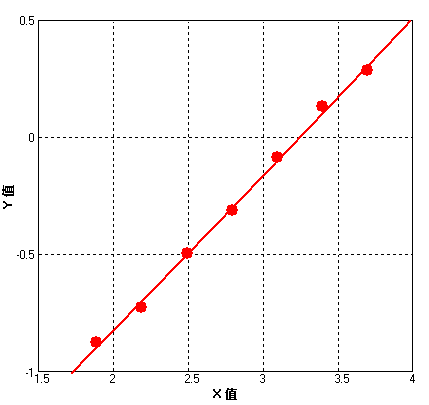

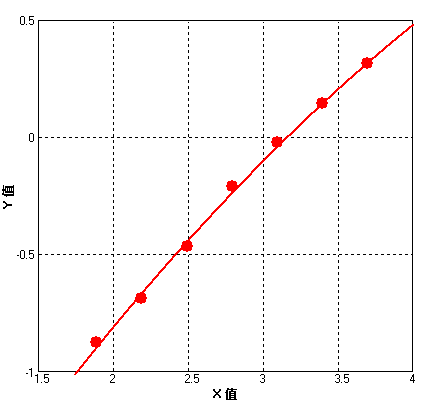

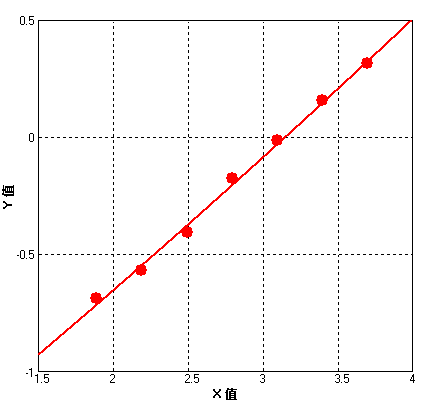


**r^2 = 0.99897**

**r^2 = 0.99819**

**r^2 = 0.99761**

**r^2 = 0.99785**

**r^2 = 0.99634**

Supplementary Figure.1. Five representative standard curves for HMGB1 detection by ELISA.

R², coefficient of determination

Supplementary Table 1. Univariate logistic regression analysis of heart failure.

| Variables | β | S.E | Z | OR(95%CI) | *P* |
| --- | --- | --- | --- | --- | --- |
| Age, years | 0.063 | 0.012 | 5.399 | 1.065 (1.041 ~ 1.089) | **<.001** |
| Male | -0.328 | 0.226 | -1.449 | 0.721 (0.463 ~ 1.122) | 0.147 |
| BMI, kg/m^2^ | -0.076 | 0.031 | -2.469 | 0.927 (0.872 ~ 0.984) | **0.014** |
| Smoking | 0.121 | 0.221 | 0.549 | 1.129 (0.732 ~ 1.740) | 0.583 |
| Drinking | -0.215 | 0.260 | -0.827 | 0.807 (0.485 ~ 1.342) | 0.408 |
| Hypertension | 0.256 | 0.221 | 1.159 | 1.292 (0.838 ~ 1.992) | 0.246 |
| Diabetes | 0.314 | 0.256 | 1.227 | 1.369 (0.829 ~ 2.262) | 0.220 |
| SBP, mmHg | 0.010 | 0.006 | 1.782 | 1.010 (0.999 ~ 1.021) | 0.075 |
| DBP, mmHg | 0.004 | 0.007 | 0.563 | 1.004 (0.991 ~ 1.017) | 0.573 |
| HR, bpm | -0.003 | 0.007 | -0.487 | 0.997 (0.983 ~ 1.010) | 0.626 |
| White blood cells , 10^9/L | -0.022 | 0.034 | -0.640 | 0.978 (0.915 ~ 1.046) | 0.522 |
| ALT, U/L | -0.011 | 0.007 | -1.635 | 0.989 (0.976 ~ 1.002) | 0.102 |
| AST, U/L | 0.017 | 0.010 | 1.808 | 1.018 (0.999 ~ 1.037) | 0.071 |
| Creatinine, μmol/L | 0.027 | 0.005 | 5.182 | 1.028 (1.017 ~ 1.038) | **<.001** |
| Uric acid, μmol/L | 0.003 | 0.001 | 2.974 | 1.003 (1.001 ~ 1.005) | **0.003** |
| CK, U/L | 0.002 | 0.001 | 1.685 | 1.002 (1.000 ~ 1.005) | 0.092 |
| CK-MB, U/L | -0.005 | 0.007 | -0.653 | 0.995 (0.982 ~ 1.009) | 0.514 |
| LDH, U/L | 0.006 | 0.002 | 2.905 | 1.006 (1.002 ~ 1.009) | **0.004** |
| SII | 0.001 | 0.000 | 3.060 | 1.001 (1.001 ~ 1.001) | **0.002** |
| SIRI | 0.170 | 0.070 | 2.412 | 1.185 (1.032 ~ 1.360) | **0.016** |
| FPG, mmol/L | 0.068 | 0.033 | 2.068 | 1.070 (1.004 ~ 1.141) | **0.039** |
| Total cholesterol , mmol/L | -0.117 | 0.097 | -1.204 | 0.890 (0.736 ~ 1.076) | 0.228 |
| Triglycerides, mmol/L | -0.114 | 0.096 | -1.179 | 0.893 (0.739 ~ 1.078) | 0.238 |
| High-density lipoprotein, mmol/L | 0.108 | 0.276 | 0.392 | 1.114 (0.648 ~ 1.915) | 0.695 |
| Low-density lipoprotein, mmol/L | -0.146 | 0.125 | -1.167 | 0.864 (0.676 ~ 1.104) | 0.243 |
| NT-pro BNP, ng/L | 0.272 | 0.064 | 4.268 | 1.313 (1.159 ~ 1.488) | **<.001** |
| LVEF, % | -0.159 | 0.026 | -6.150 | 0.853 (0.811 ~ 0.897) | **<.001** |
| LAD, mm | 0.148 | 0.024 | 6.058 | 1.159 (1.105 ~ 1.216) | **<.001** |
| LVEDD, mm | 0.118 | 0.022 | 5.275 | 1.125 (1.077 ~ 1.176) | **<.001** |
| HMGB1,ng/ml | 1.079 | 0.169 | 6.393 | 2.942 (2.113 ~ 4.095) | **<.001** |
| HMGB1 Tertile |  |  |  |  |  |
| T 1 |  |  |  | 1.000 (Reference) |  |
| T 2 | 1.024 | 0.297 | 3.445 | 2.784 (1.555 ~ 4.985) | **<.001** |
| T 3 | 2.095 | 0.306 | 6.856 | 8.125 (4.464 ~ 14.789) | **<.001** |

T1: 0<HMGB1≤0.896ng/mL; T2: 0.896ng/mL<HMGB1≤1.472ng/mL; T3 :HMGB1>1.472ng/mL BMI,Body Mass Index; HR,Heart Rate; SBP,Systolic Blood Pressure; DBP,Diastolic Blood Pressure; ACEI,angiotensin converting enzyme inhibitor; ARB, angiotensin receptor blocker; CCB, Calcium channel blockers; FPG,fasting plasma glucose; ALT, alanine amino transferase; AST,aspartate amino transferase; CK,creatine kinase; CK-MB,creatine kinase-myocardial Band; LDH: lactate dehydrogenase; SII,Systemic immune-inflammation index; SIRI,system inflammation response index; LVEF,left ventricular ejection fraction; LAD,left atrial diameter; LVEDD,left ventricular end-diastolic diameter.
